# Multi-year predictability of climate, drought, and wildfire in southwestern North America

**DOI:** 10.1038/s41598-017-06869-7

**Published:** 2017-07-26

**Authors:** Yoshimitsu Chikamoto, Axel Timmermann, Matthew J. Widlansky, Magdalena A. Balmaseda, Lowell Stott

**Affiliations:** 1 Department of Plants, Soils and Climate, Utah State University, Logan, Utah USA; 2 Institute for Basic Science Center for Climate Physics, Pusan National University, Busan, South Korea; 3 Joint Institute for Marine and Atmospheric Research, University of Hawaii at Manoa, Honolulu, Hawaii USA; 4 European Centre for Medium-Range Weather Forecasts, Reading, UK; 5 Department of Earth Sciences, University of Southern California, Los Angeles, California, USA

## Abstract

Past severe droughts over North America have led to massive water shortages and increases in wildfire frequency. Triggering sources for multi-year droughts in this region include randomly occurring atmospheric blocking patterns, ocean impacts on atmospheric circulation, and climate’s response to anthropogenic radiative forcings. A combination of these sources translates into a difficulty to predict the onset and length of such droughts on multi-year timescales. Here we present results from a new multi-year dynamical prediction system that exhibits a high degree of skill in forecasting wildfire probabilities and drought for 10–23 and 10–45 months lead time, which extends far beyond the current seasonal prediction activities for southwestern North America. Using a state-of-the-art earth system model along with 3-dimensional ocean data assimilation and by prescribing the external radiative forcings, this system simulates the observed low-frequency variability of precipitation, soil water, and wildfire probabilities in close agreement with observational records and reanalysis data. The underlying source of multi-year predictability can be traced back to variations of the Atlantic/Pacific sea surface temperature gradient, external radiative forcings, and the low-pass filtering characteristics of soils.

## Introduction

Over the past six years, several regions in North America have experienced severe drought conditions. Their impacts cover a wide range of sectors such as agriculture, energy, food security, forestry, drinking water, and tourism^1, 2^. Unusually dry and hot conditions were reported for Texas and Mexico in 2010–2011^3^, for the Great Plains in 2012^4^, and for California in 2011–2014^5, 6^. The economic damage associated just with the recent California drought has been estimated at ~2.2 billion United States (US) Dollars and a loss of ~17,000 jobs^7^. Successful water resource management that relies on knowledge of the present and future hydroclimatic conditions is crucial for mitigating the climate-driven drought risks.

Even though the atmosphere has a short dynamical memory of less than several weeks, its evolution is partly affected by slowly varying sea surface temperature (SST) conditions. In the low-frequency range, atmospheric variability is modulated by more predictable climate phenomena such as the El Niño-Southern Oscillation (ENSO)^8^, the Pacific Decadal Oscillation (PDO)^9^, the Atlantic/Pacific SST contrast^10, 11^, and the Atlantic Multidecadal Oscillation^12, 13^. The ratio of internally generated atmospheric variability and SST-forced variability thus limits the potential prediction horizon of monthly to seasonally averaged rainfall changes to less than 1 year^14–16^. However, there are many land systems (e.g., soils, water reservoirs, vegetation, and perennial snowpack) that effectively filter out the high-frequency rainfall variability and therefore exhibit longer persistence as a result of natural time integration of atmospheric signals^17, 18^. Consequently, this low-pass filtering effect enhances the contribution of the SST-forced component to hydroclimate variability^16^ and the predictability-relevant signal-to-noise ratio^19^. Moreover, changes in the hydrological cycle due to increasing anthropogenic radiative forcings have been suggested as contributors to recent multi-year drought events^20, 21^.

Here we determine the impacts of ocean variability and external radiative forcings (following the RCP4.5 forcing scenario) on climate, drought, and wildfire probability over southwestern North America. To disentangle their relative contributions and interactions, we use the fully coupled Community Earth System Model (CESM) in three experiments (see Methods): an uninitialized forced run (**UR**), an ocean data assimilation run (**AR**), and a series of multi-year initialized prediction runs (**IR**). Whereas the **UR** captures the climate response to external radiative forcings (natural and anthropogenic), the **AR** represents combined ocean and radiative impacts. The resulting **AR** simulates climatology and variability of annual mean precipitation over North America in close agreement with observations (Fig. S1). Predictability of fire probability and soil moisture is determined by dynamical hindcast/forecast experiments in **IR**.

## Results

### Coherent variations of climate, drought, and wildfire probability

**Figure 1:**
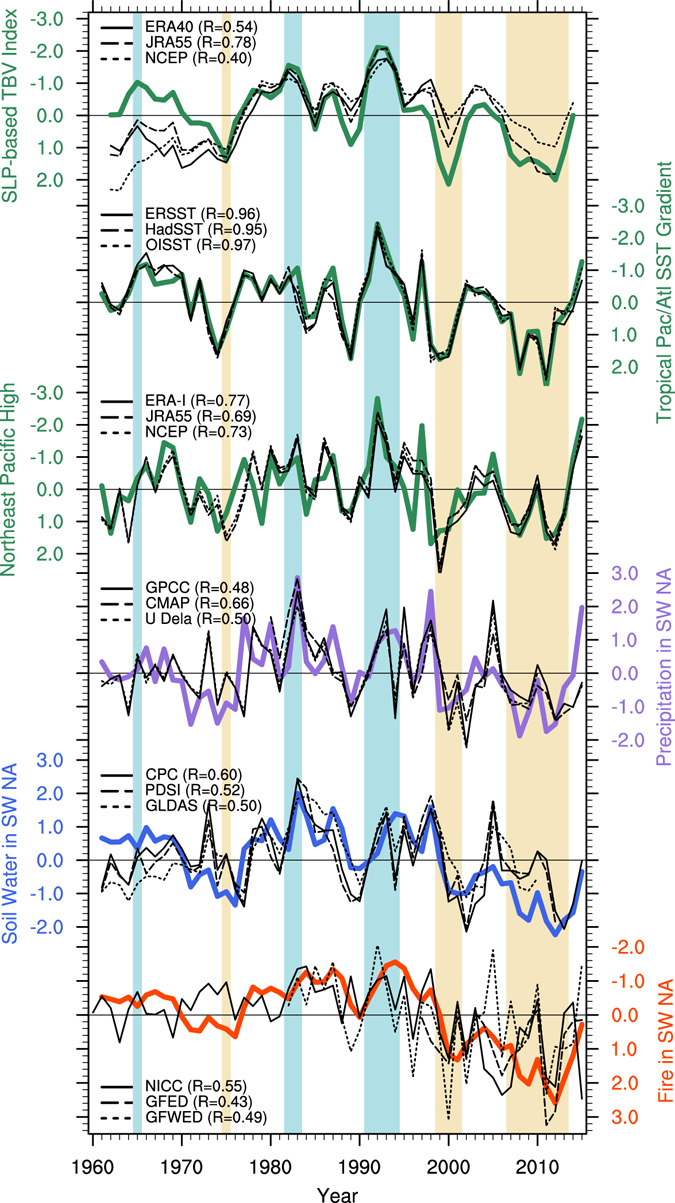
Water year (October of the previous year to September) normalized time series of SLP-based trans-basin variability (TBV) index (top; the Principal Component of the first EOF mode of 3-year running mean filtered SLP anomalies^10^ from 60°S–60°N; reversed Y-axis, a positive index refers to anomalously low (high) SLP over the equatorial Atlantic (Pacific).), tropical Atlantic/Pacific SST gradient (second; standardized SST anomaly difference between the tropical Atlantic-Indian Ocean and the tropical central Pacific; reversed Y-axis), Northeast Pacific High anomalies (third; SLP anomalies averaged over 20°N–35°N, 150°W–120°W; reversed Y-axis), and the southwestern North American (SW NA) hydroclimate anomalies (averaged over 28°N–44°N, 125°W–100°W; black box in Fig. S3d) of precipitation (fourth; defined as precipitation anomaly divided by climatological mean), soil water (fifth; averaged from surface to 3-m depth), and wildfire (bottom panels; reversed Y-axis) in observation-based datasets (black; see Supplementary Methods with references) and assimilation run (**AR**) (colored lines). Correlation coefficients between observational estimates and **AR** are denoted in brackets. Shaded beige and cyan regions indicate water years with +/− one standard deviation in the TBV index. Anomalies are defined as a deviation from 1960–2015 climatological mean in **AR**. Climatological means, anomalies, and standard deviations in observational estimates are adjusted to have the same reference period as **AR** based on the available period of each observational estimates (see Supplementary Methods). Plots were generated using the NCAR Command Language (Version 6.3.0) [Software]. (2016). Boulder, Colorado: UCAR/NCAR/CISL/TDD. http://dx.doi.org/10.5065/D6WD3XH5.

The **AR** simulation reveals a tight connection between changes of the tropical Atlantic/Pacific SST gradient, the global Walker circulation, atmospheric pressure in the northeastern Pacific, and hydroclimate conditions over the southwestern part of North America (Fig. 1). More specifically, we see that assimilated oceanic SST anomalies are sufficient to simulate realistic trans-basin sea level pressure (SLP) variability. Associated with the phase of the Atlantic/Pacific trans-basin variability (TBV) SLP index (see Methods), we find coherent temporal evolutions in precipitation and soil water averaged over southwestern North America (28°N–44°N, 125°W–100°W). These simulated anomalies in water year basis are in good agreement with observations and reanalysis datasets for 1960–2015 (correlation coefficients, R ≥ 0.48), even though our assimilation system does not include any atmospheric or land observations. The consistency is further emphasized for the respective low-frequency components (R ≥ 0.54; Fig. S2), indicating strong SST impacts on hydroclimate anomalies in southwestern North America. The robust relationship between TBV and North American drought conditions is further illustrated by comparing the TBV index with the results of a Singular Value Decomposition (SVD) analysis of observed and simulated (ensemble mean of **AR**) precipitation and soil water anomalies (Fig. S3e,f). The SVD analysis also reveals prominent coherence between observed and simulated hydroclimate climate variations in southwestern North America and somewhat weaker reproducibility in the northeastern part of US.

In addition to the hydroclimate variations, our model simulates the annual fire season length in reasonable agreement with the observational estimates of the burned area and the fire weather index (R = 0.43–0.55 in Fig. 1), despite the fact that the fire parameterization in this model is quite simple and observational estimates of fire products are limited in terms of geographic representation (the federal reports cover only the US boundary), temporal coverages (the satellite measurement is available after 1991), and fire-related variables (the burned area or fire weather index; see Supplementary Methods). In the model, the annual fire season length is calculated from the annual fractional area burned, using an e-folding approximation for the sum of daily fire occurrence probabilities over one year^22, 23^ (see Methods). Because of this parameterization, the model simulated fire season length (i.e., annual fire probability) is highly correlated with total soil water variability in southwestern North America (R = −0.93; blue and red lines in Fig. 1). The significant correlations between the model simulated fire probability and the observational estimates of different fire products imply that the large-scale wildfire activity can be explained by the atmospheric response to the low-frequency ocean SST anomalies assimilated in **AR**. Our results are also consistent with previous observational findings that show the close relationship between the hydroclimate conditions and annual burned area in southwestern North America^24, 25^.

### Sources of multi-year predictability

The annual mean SLP variability associated with TBV affects North American hydroclimate variability through wind and moisture transport changes. A positive Atlantic/Pacific SST gradient leads to anomalous low pressure over the tropical Atlantic and high pressure over the Pacific (Fig. 1), which includes an anomalous high-pressure ridge in the northeastern Pacific (Fig. S4, right panel) and a northward shift of the jet stream^16^. Northeasterly wind anomalies associated with this high-pressure ridge reduce moisture transport from ocean to continent and cause anomalously dry conditions in North America, as exemplified by the recent multi-year California drought^6, 26^, which occurred during an extreme positive phase of the TBV (Fig. 1). Similar patterns, but with opposite signs, are found during the negative TBV phase composites (Fig. S4, left column). The importance of the Northeast Pacific High for droughts in the southwestern part of North America and the 2011–2014 California drought event, in particular, is well established^4, 6, 15^. However, the origin of this circulation feature and its low-frequency modulation still remain elusive^27^. Previous studies have documented influences of SST anomalies in the equatorial^6, 26, 28^ and the western tropical Pacific^29^, the Kuroshio-Oyashio region^30^, the Atlantic Ocean^12, 31^, and the Indian Ocean^32^. Using the CESM, our results clearly document that the Atlantic/Pacific trans-basin SST gradient and the corresponding global shifts of the Walker Circulation are key drivers for the multi-year to decadal changes in northeastern Pacific SLP and North American hydroclimate (Figs 1 and S3).

**Figure 2:**
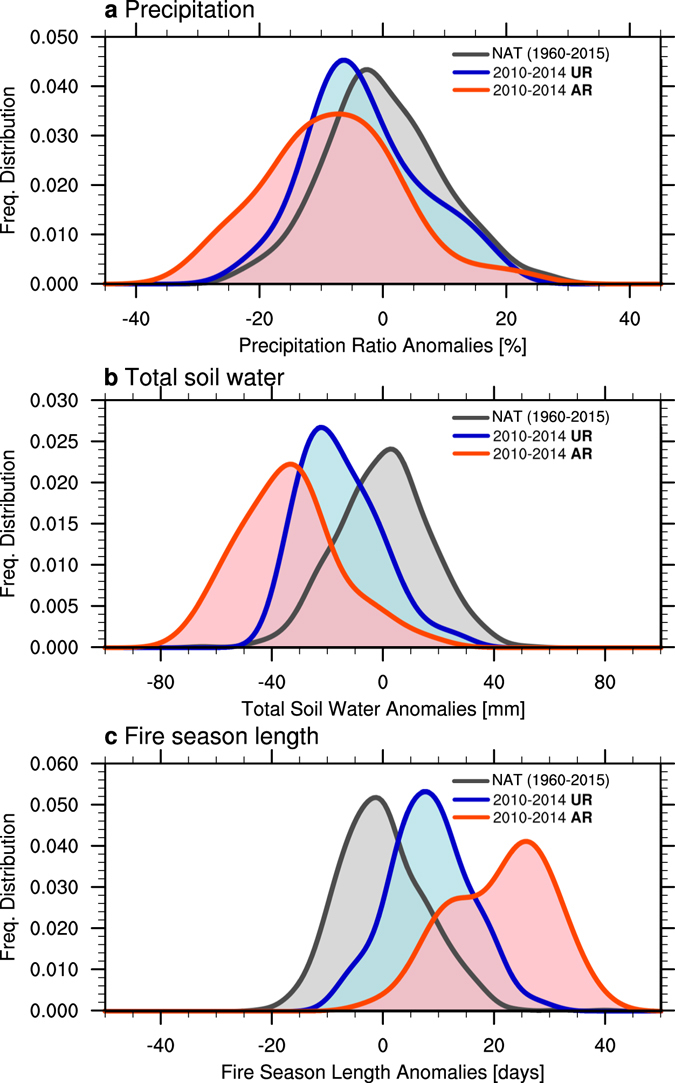
Frequency distributions of simulated (**a**) precipitation (defined as precipitation anomaly divided by climatological mean), (**b**) total soil water, and (**c**) fire season length anomalies averaged over southwestern North America (28°N–44°N, 125°W–100°W) for the natural (i.e., unforced) variability during 1960–2015 (black) and the external (blue) and the ocean + external components during the period 2010–2014 (red). The frequency distribution of natural variability (NAT) is determined by calculating the water year deviations of individual ensemble members from the ensemble mean in **UR** for 1960–2015 (i.e., ensemble spread in the uninitialized run). The frequency distributions of external and ocean + external components are estimated from the water year anomalies of individual ensemble members during 2010–2014 in **UR** and **AR**, respectively (i.e., 50 samples in each). A Kernel smoothing function is applied to these distributions. Plots were generated using the NCAR Command Language (Version 6.3.0) [Software]. (2016). Boulder, Colorado: UCAR/NCAR/CISL/TDD. http://dx.doi.org/10.5065/D6WD3XH5.

In addition to the direct impact of global SST anomalies on North American rainfall patterns, radiative forcings may have also contributed to recent decadal drought and wildfire trends^33, 34^. A previous study suggested that greenhouse warming may have played a role in the Central America/Mexico region^35^ and the 2011–2014 California drought event^6, 36^. This is further supported by our analysis of the aggregated frequency distributions of precipitation, soil water conditions, and annual fire season length for the simulated 2010–2014 period in the externally forced 10-member ensemble of the **UR** experiments and assimilation runs **AR** (Fig. 2). Compared to the frequency distribution of natural variability for the 1960–2015 period, we see a shift of the distribution towards drier conditions and increased wildfire season length during the 2010–2014 segment (Fig. 2), if the external forcing is included in **UR** (blue), and an additional amplification of this effect if observed SST anomalies are considered (**AR**, red). In other words, severe droughts and increased wildfire probabilities during 2010–2014 were likely caused by a combination of decadal climate conditions associated with the positive phase of TBV (Fig. 1) and the direct climate response to the natural and anthropogenic radiative forcings. In terms of the changes in the mode of frequency distribution, the drier soil water conditions are caused by the comparable contributions from the ocean anomalies and the externally forced components, whereas the ocean component contributes to the increased fire probability twice as much as to the externally forced component. Nevertheless, both processes could contribute to the multi-year drought predictability beyond the atmospheric short-term memory.

An additional source of multi-year drought predictability is associated with the low-pass filtering characteristics of soil water content relative to the more unpredictable precipitation variations^16^. This is clearly illustrated in the **AR** simulation with pronounced precipitation spikes in southwestern North America in 1977, 1983, 1998, and 2008 and much smoother soil water variability (Figs 1 and S3). In fact, the shift of the frequency distribution during the 2010–2014 period relative to the natural variability is much larger for soil water anomalies as compared to the precipitation (Fig. 2). Moreover, the filtering effect of soils induces a delayed soil water response in relation to the SST forcings, which is confirmed by a lag correlation between the TBV index and soil water anomalies in southwestern North America in Fig. 1 (the correlation coefficient becomes maximum when the former leads the later at one year). These two processes (i.e., the filtering effect and the delayed response) contribute to the long-term multi-year hydroclimate predictability.

### Impacts of the tropical trans-basin variability on drought/fire conditions

**Figure 3:**
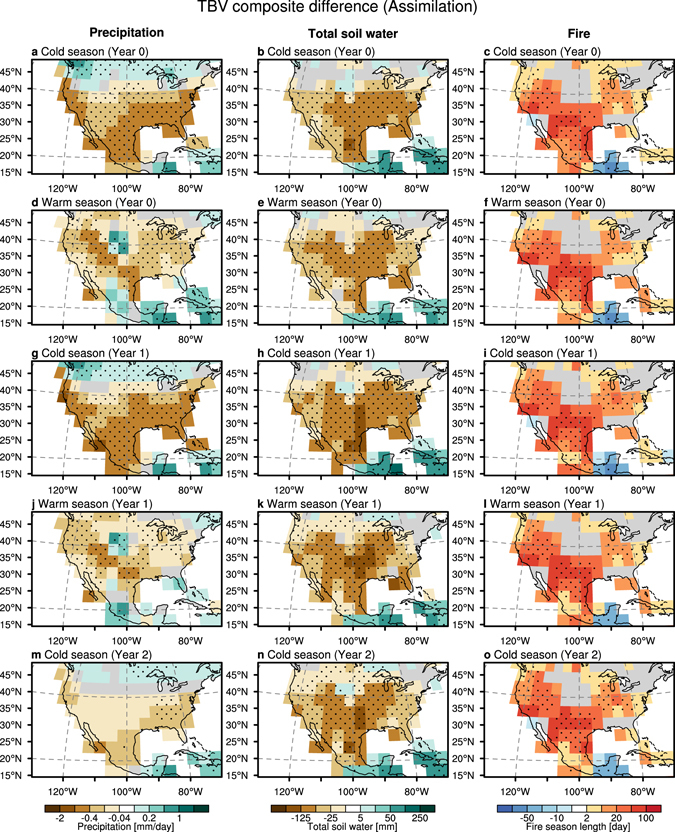
Temporal evolution of composite anomalies in precipitation (left), total soil water (middle), and fire season length (right) in (**a**–**c**) cold and (**d**–**f**) warm seasons at lag 0-year, (**g**–**i**) cold and (**j**–**l**) warm seasons at lag 1-year, and (**m**–**o**) cold season at lag 2-year, associated with the difference between 11 positive and 7 negative years of TBV phases (see Methods) in **AR**. Dotted areas indicate the statistically significance at 90% level on the basis of two-side Student’s t-test. The region with climatologically low fire activity is masked out (less than 7 days of fire season length per month). Plots were generated using the NCAR Command Language (Version 6.3.0) [Software]. (2016). Boulder, Colorado: UCAR/NCAR/CISL/TDD. http://dx.doi.org/10.5065/D6WD3XH5.

**Figure 4:**
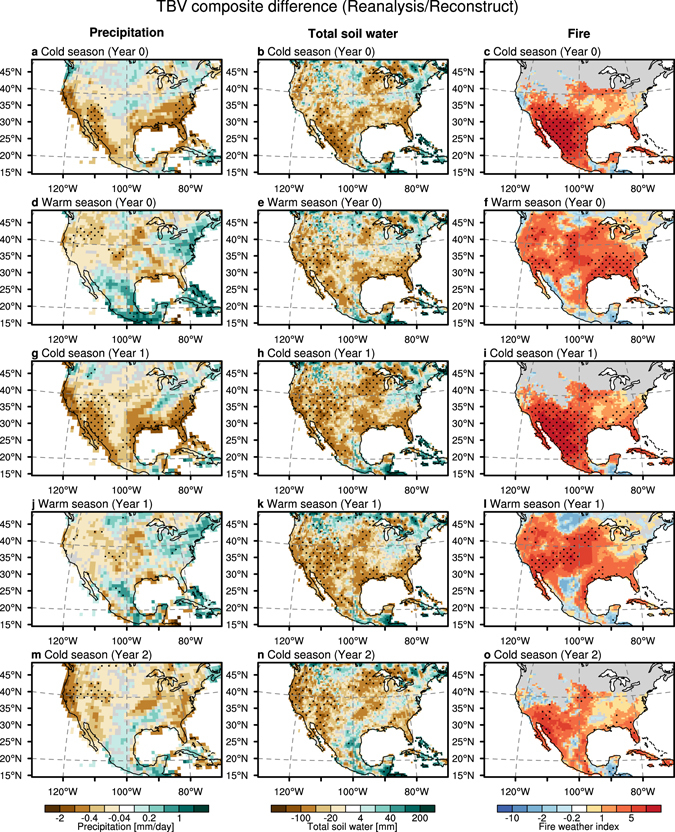
Same as Fig. 3, but for observational estimates for precipitation in GPCC, total soil water in CPC, and fire weather index in GFWED (see Supplementary Methods). The composite difference of fire weather index is evaluated by 10 positive and 6 negative years because of the available data length. The region with climatologically low fire activity is masked out (less than 1 fire weather index per month). Plots were generated using the NCAR Command Language (Version 6.3.0) [Software]. (2016). Boulder, Colorado: UCAR/NCAR/CISL/TDD. http://dx.doi.org/10.5065/D6WD3XH5.

The ocean impact on drought/fire conditions over North America is further illustrated by composite analyses for extreme phases of the TBV index. When the eastern tropical Pacific is cooler than the tropical Atlantic (i.e., for the positive TBV phase), the **AR** simulation shows negative precipitation anomalies south of 40°N during the cold season (October–March; Fig. 3a) and their northward shift over the western part of North America during the warm season (April–September; Fig. 3d). These precipitation patterns are still prevalent for the respective seasons 1 year after the mature stage of TBV. Two years later, however, their signal vanishes and becomes statistically indistinguishable from white noise for southwestern North America (left panels in Fig. 3). In contrast to the precipitation response, the total soil water anomalies exhibit less seasonality and longer memory: the dry conditions over the entire North America persist in all seasons for 0–2 years lag (center panels in Fig. 3). These characteristics are also confirmed in the observational estimates (Fig. 4), albeit with reduced statistical significance compared to the **AR** simulations. Because of the strong linkage to soil moisture, the model simulated fire probability shows a similar response to the soil water anomalies (right panels in Fig. 3). The consistent fire changes are also captured by the observation-based fire weather index, particularly over the southwestern part of North America, although there is a discrepancy between model and observation in the northwestern part of US during the cold season (right panels in Fig. 4). These results also support that the low-frequency Atlantic/Pacific SST gradient variability contributes to the multi-year potential predictability of drought-fire conditions over southwestern North America.

### Multi-year predictions of drought-fire conditions over southwestern North America

**Figure 5:**
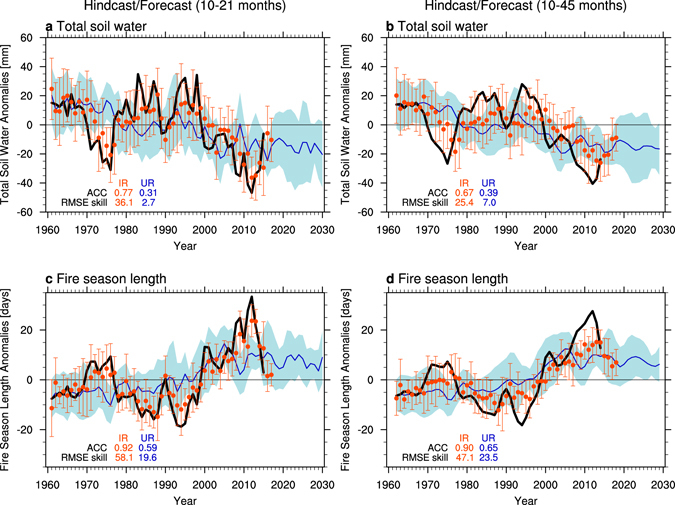
Predictability of total soil water (top) and fire season length anomalies (bottom) for 10–21 (left; annual water year from October to September) and 10–45 months lead time (right; 3 water years) over the southwestern North America (28°N–44°N, 125°W–100°W) in the **IR** (red; ensemble mean in circle and its spread in error bars), **UR** for 1961–2030 (blue; ensemble mean for line and its spread for shading), and **AR** for 1961–2015 (black). Anomaly correlation coefficients (ACC) and root-mean-squared-error (RMSE) skill (%) in **IR** (red) and **UR** (blue) against the **AR** are denoted in each panel. The latest forecast in **IR** is initialized in January 1st, 2016 and represents the forecasted anomalies for the water year 2017 in left and 2017–2019 in right panels. Plots were generated using the NCAR Command Language (Version 6.3.0) [Software]. (2016). Boulder, Colorado: UCAR/NCAR/CISL/TDD. http://dx.doi.org/10.5065/D6WD3XH5.

**Figure 6:**
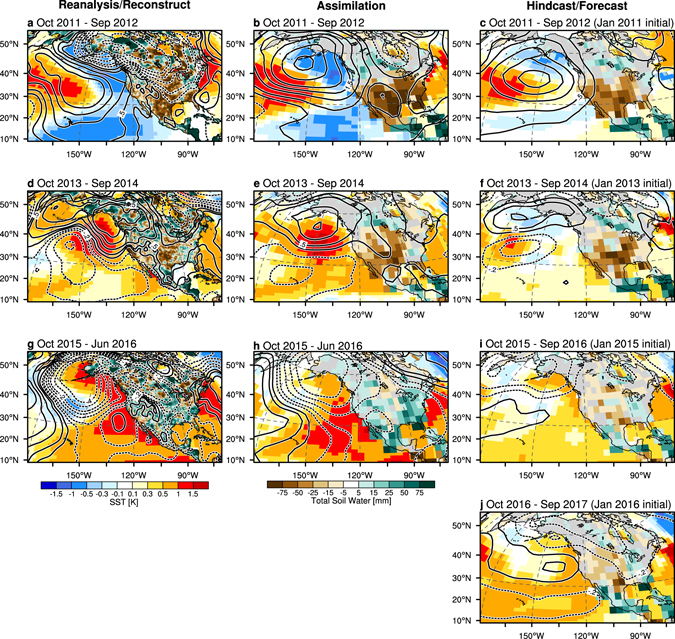
Anomaly maps of SST (shaded ocean), SLP (contour), and total soil water content (shaded land) for the water years (**a**–**c**) 2012, (**d**–**f**) 2014, (**g**–**i**) 2016, and (**j**) 2017 in reanalysis/observational datasets (left), **AR** (center; ensemble mean), and **IR** (right panels; ensemble mean of 10–21 months lead time predictions initialized in January of the year preceding the water year). Reanalysis datasets of SST, SLP, and total soil water anomalies are obtained from ERSST, ERA-I, and CPC, respectively (see Supplementary Methods). Anomalies are defined as the deviation from the climatological mean for 1990–2009. The water year 2016 in (**g**) reanalysis/observational datasets and (**h**) **AR** is verified by 9-month average (October 2015 to June 2016). Predictions are initialized in January 1st of (**c**) 2011, (**f**) 2013, (**i**) 2015, and (**j**) 2016. Contour intervals for SLP anomalies are ±0.1, ±0.2, ±0.5, ±1.0, ±1.5, ±2, ±3, and ±4 Pa. Plots were generated using the NCAR Command Language (Version 6.3.0) [Software]. (2016). Boulder, Colorado: UCAR/NCAR/CISL/TDD. http://dx.doi.org/10.5065/D6WD3XH5.

The ensemble mean of our initialized CESM hindcast experiments (**IR**) documents a high level of multi-year predictability for soil water content and fire season length over southwestern North America (Figs 5, 6 and S5). These predictions are initialized once a year on January 1^st^ using the global ocean conditions from **AR** as well as the external forcings due to increasing greenhouse gas concentrations and land use change. Our 10–21 month lead time forecasts for soil water content and fire season length (for water year mean anomalies averaged from October of the previous year to September) and the corresponding ensemble spread (i.e., **IR**) align well with those simulated in **AR** (Fig. 5) and the observational estimates (Fig. 1). According to our forced simulations (**UR**), external forcing has contributed to the recent long-term trends of soil water anomalies and an extension of the fire season length (blue lines in Fig. 5). Although there is some suggestion for a saturation of this contribution around 2020 in the RCP4.5 radiative forcing scenario, the long-term trends still continue in the RCP4.5 or RCP8.5 scenarios, at least until 2100 (Fig. S6). In addition to the external radiative forcing, observed SST variability has further exacerbated these trends since the late 1990s (black line in Fig. 5). The largest anomalies of soil water and fire probability occurred during the positive TBV phase from 2010 to 2014 (Figs 1 and 5). Our 10–21 month and 10–45 month predictions capture this temporal evolution well (red circles in Fig. 5), albeit with an underestimation in amplitude.

Our finding of multi-year drought predictability and the understanding of the underlying processes provides the rationale to conduct long-term drought predictions, beyond the seasonal timescale. In the past decades, the advanced understandings of drought dynamics over western North America^6, 26^ have contributed to developing the drought predictions particularly on seasonal timescales^37, 38^. In contrast to other seasonal prediction systems, which suffer from artificial climate drifts during a multi-seasonal prediction^39^, our new CESM prediction system shows negligible model drift during the prediction. This enables us to disentangle the low-frequency ocean impacts from the effects of radiative forcings. Whereas our study focuses on the multi-year drought events after 1960, the dynamical process associated with TBV is consistent with the previous atmospheric modeling studies in which the past multi-year drought events (like the Dust Bowl during the 1930s) have been attributed to La Niña-like conditions in the Pacific and warm conditions in the subtropical North Atlantic Ocean^40–42^. In contrast to the strong seasonality of ENSO, the tropical SST gradient between Pacific and Atlantic associated with TBV can modulate the North Pacific SLP variability for all seasons (Fig. S7). This year-around impact of TBV contributes to the enhanced predictability, beyond the typical predictable limit of ENSO. The predictive skills of drought/fire conditions in our system degrades after about 2-years lead time (Fig. S8).

Our forecasts for the water year 2017 (October 2016 to September 2017) show a demise of the previous drought conditions and a subsequent transition to normal soil water and wildfire conditions in the southwestern North America (Figs 5 and 6). For the water year 2012, observations and **AR** show the typical SLP and SST anomalies patterns related to a negative phase of PDO: anomalous high pressure over extratropical North Pacific and warm surface waters in the extratropical western Pacific surrounded by cold ones in the east (Fig. 6a,b). These atmospheric anomalies contribute to the anomalous high-pressure ridge in the northeastern Pacific, which in turn generates dry conditions in the southwestern part of North America where soil water variability has multi-year predictive skill (Fig. S5). Whereas SST anomalies tend to switch their sign from 2012 to 2014, positive SLP anomalies and dry soil conditions still persist in the higher latitude of the northeastern Pacific and Southwestern US/Mexico regions, respectively (Fig. 6d,e). The ensemble means of 10–21 month lead time predictions capture these large-scale SLP and soil water anomaly patterns in 2012 and 2014 (Fig. 6c–f) well. In the water year 2016, however, the TBV shows its phase change associated with the 2015/16 El Niño event (Fig. 6g–i), which contributes to a subsequent establishment of a High-pressure ridge over the eastern North Pacific. Because of the typical ENSO skill loss after the spring^43^, the active 2015/16 El Niño event may cause the large uncertainty of TBV prediction for the 2017 water year.

## Discussion

This study revealed that the Atlantic/Pacific Ocean SST and SLP contrast is one of the main drivers for low-frequency variability in soil water and fire probability in southwestern North America. From a realistic coupled model assimilation run, as well as using forecasts initialized from this experiment, we find clear evidence for multi-year predictive skills of drought-fire conditions in the southwestern part of North America (Fig. S5). Soils effectively filter out unpredictable high-frequency variations in precipitation, leaving a longer-term memory^16, 44^, and a higher correlation with low-frequency multi-year SST variability. Utilizing this source of predictability and combining with the current seasonal prediction products, our initialized forecasts could help in determining regional climate risks over North America with potential benefits for agriculture, water management, and forestry.

Whereas our approach demonstrates how low-frequency ocean variability affects simulated wildfire probabilities in southwestern North America, there is still a large gap in our understanding of how large-scale climate anomalies influence local-scale wildfires. On a regional scale, the climate-fire relationship depends strongly on the prevailing vegetation type. In forested systems, drought can enhance wildfire probabilities, whereas non-forested systems experience a reduction of fire probability due to a reduction in fuel associated with drought events^24, 25, 45^. Our model results suggest that the low-frequency climate impact on wildfire probabilities is more pronounced in forested systems compared to rangeland systems. However, the detailed landscape characteristics in forested and semi-arid systems and their different responses to climate are not adequately resolved in our model, because the vegetation parameterization of the land model uses plant function types. Moreover, the fire probability in our model shows less seasonality compared to the observations. To further improve the climate-fire predictability, it is crucial to get a better understanding of seasonal variations in TBV, regional climate and fire probability. This issue could be particularly relevant for US montane regions^45^ where wildfire risk is strongly impacted by changes in seasonal snowpack. Future analysis of observational fire datasets^46, 47^ will be helpful in examining the regional drought-fire relationships as well as to improve the parameterizations of fire probabilities in climate models.

## Methods

### Model and Experiments

The coupled general circulation model (CGCM) adopted here is a lower-resolution version of the community earth system model CESM 1.0.3^48^. The atmospheric model uses a T31 horizontal spectral truncation (approximately 3.75° horizontal resolution) with 26 hybrid sigma/pressure coordinate levels. The land model has the same horizontal resolution as the atmospheric component and uses 10 soil layers and aquifer water with changing water table depth^49^. The ocean component uses a displaced North Pole, 3° horizontal resolution and 60 vertical levels. No flux correction is applied in exchanging heat, water, and momentum fluxes between the atmosphere and the ocean.

Using CESM, we conducted three experiments: an uninitialized forced run (**UR**) covering the period from 1850–2030, an assimilation run (**AR**) for the period of 1960–2015, and a series of multi-year initialized prediction runs (**IR**), which are initialized once a year from the **AR** (see Supplementary Methods). The climate response to external radiative forcings (natural and anthropogenic) is estimated from a 10-member ensemble mean of forced historical simulations with CESM (**UR**). Combined ocean and radiative impacts are evaluated by assimilating observation-based 3-dimensional ocean temperature and salinity anomalies from January 1958 to June 2016^50^ into CESM (**AR**) and by prescribing the same radiative forcing as in **UR**. By initialized every year from **AR**, we conduct a series of 10-year-long hindcast/forecast CESM experiments (**IR**). The latest **IR** forecast is initialized using the observed ocean anomaly conditions of January 1st, 2016. In all experiments, we conduct a 10-member ensemble simulation for individual runs and use historical observed data of natural and anthropogenic forcing (greenhouse gas and aerosol concentrations, solar cycle variations, and major volcanic eruptions) before 2005 and the IPCC RCP4.5 future emissions scenario from 2005.

### Wildfire probability

Wildfire probability is evaluated by annual fire season length in the model. In the fire scheme of CESM 1.0.3, the annual fire season length is estimated by an e-folding approximation for the annual sum of a daily probability of fire occurrence at least once a day. This daily fire probability in each grid box is parameterized in terms of fuel density obtained from the vegetation carbon, upper soil water content, and surface air temperature. The sum of daily fire probability over the whole year corresponds to the annual fire season length in the model. This fire scheme simply assumes that the annual fractional burned area is exponentially increased associated with increase in the annual fire season length, which is verified by observational reports. Because of this exponential behavior and the lower horizontal resolution in our land model, the area average of model simulated burned area is highly biased only by a few grid points. On the other hand, the annual fire season length shows more linear characteristics compared to the exponential behavior of burned area. As a result, the area average of annual fire season length reflects on the large-scale fire probability whereas that of annual burned area is affected by a local-scale and individual large fire event. The detail of fire scheme is described in previous studies^22, 23^.

### TBV index

The tropical trans-basin variability (TBV) index is defined as the principal component time series of the first EOF mode for 3-year running mean filtered SLP variability in 60°S–60°N. A positive TBV index corresponds to the warm SST/low SLP over the tropical Atlantic but cold SST/high SLP in the tropical central Pacific, and vice versa (Fig. S4). To follow the phase of SLP-based TBV index, the tropical Atlantic/Pacific SST gradient is obtained from the normalized SST anomalies averaged over the tropical Atlantic-Indian Ocean (15°S–15°N, 40°W–60°E) minus those in the tropical central Pacific (15°S–15°N, 180°W–150°W) as shown by previous study^10^. From the SLP-based TBV index, 11 positive (7 negative) years are identified as 1975, 1999, 2000, 2001, 2007, 2008, 2009, 2010, 2011, 2012, and 2013 (1965, 1982, 1983, 1991, 1992, 1993, and 1994).

## Electronic supplementary material

Supplementary Information
