# Supplementary material for: Multi-year predictability of climate, drought, and wildfire in southwestern North America
Source: Sci Rep. 2017 Jul 26;7:6568. doi: 10.1038/s41598-017-06869-7 (PMC5529505; doi:10.1038/s41598-017-06869-7)
Supplement: Supplementary file 1 — Supplementary Information [file 41598_2017_6869_MOESM1_ESM.pdf]

Supplementary Information, to "Multi-year predictability of climate, drought, and wildfire in southwestern North America"

Yoshimitsu Chikamoto[1]*, Axel Timmermann[2], Matthew J. Widlansky[3], Magdalena A. Balmaseda[4], and Lowell Stott[5]

[1]Department of Plants, Soils and Climate, Utah State University, Logan, Utah, USA.

[2]Institute for Basic Science Center for Climate Physics, Pusan National University, Busan, South Korea.

[3]Joint Institute for Marine and Atmospheric Research, University of Hawaii at Manoa, Honolulu, Hawaii, USA.

[4]European Centre for Medium-Range Weather Forecasts, Reading, UK.

[5]Department of Earth Sciences, University of Southern California, Los Angeles, California, USA

Supplementary Methods:

Observational datasets

To verify the model simulations, we use multiple observational reconstructions and reanalysis datasets. Precipitation and soil water observations with the 1° latitude-longitude grid from January 1960 to June 2016 are used from Global Precipitation Climatology Centre (GPCC) combined full version 7 and version 4 monitoring data products[1] and National Oceanic and Atmospheric Administration (NOAA) Climate Prediction Center (CPC) reanalysis product[2], respectively. To assess the observed variability of precipitation and soil water over southwestern North America, we additionally use the CPC Merged Analysis of Precipitation (CMAP) for 1979–2015[3], the University of Delaware (U Dela) monthly precipitation version 3.01 for 1960–2010[4], the monthly self-calibrated Palmer Drought Severity Index (PDSI) for 1960–2012[5], and the total soil water (upper 2 m) in the Global Land Data Assimilation System (GLDAS) with Noah land surface model for 1960–2010[6]. Observed SLP-based TBV indices are obtained from the first leading EOF mode of 3-year running mean filtered SLP anomalies within 60°S–60°N using the European Centre for Medium-Range Weather Forecasts (ECMWF) 40-year reanalysis dataset (ERA40) for 1960–2001[7], the Japanese 55-year Reanalysis (JRA55) for 1960–2013[8], and the National Centers for Environmental Prediction (NCEP) and the National Center for Atmospheric Research (NCAR) Reanalysis for 1960–2015[9]. Northeastern Pacific High is calculated from SLP anomalies averaged over 20°N–35°N, 150°W–120°W in ECMWF Reanalysis Interim (ERA-I) for from January 1979 to June 2016[10] as well as JRA55 and NCEP. The ERA-I and NCEP reanalysis datasets are also used to make observed SLP anomaly maps in Figs. 4 and S4, respectively. Tropical Pacific/Atlantic SST gradients are estimated by the zonal gradient of standardized SST anomalies between the tropical central Pacific (15°S–15°N, 180°–150°W) and the tropical Atlantic-Indian Ocean (15°S–15°N, 40°W–60°E) using Extended Reconstructed Sea Surface Temperature (ERSST) version 4 from January 1960 to June 2016[11], Hadley Centre SST data set (HadSST) version 3.1.1.0 for 1960–2014[12], and NOAA Optimum Interpolation Sea Surface Temperature (OISST) version 2 for 1982–2015[13]. Southwestern North America wildfire observations are obtained from the annual burned area reported by the National Interagency

Coordination Center (NICC) for 1960–2015 (only covered over the United States)[14], the satellite-based monthly burned area without small fires in Global Fire Emissions Database version 4 (GFED4) for 1996–2015[15], and the monthly fire weather index estimated by the NASA Modern Era Retrospective Analysis for Research and Applications version 2 in Global Fire Weather Database (GFWED) for 1981–2015[16].

Normalized time series of observational estimates in Figure 1 are obtained in the following steps. First, climatological mean, anomaly from it, and standard deviation in observational estimate are calculated based on the available period in each record. Next, the anomalies in observational estimate are normalized by its standard deviation with a factor that corresponds to the ratio of standard deviations in assimilation run **AR** during the observational period and the all assimilation period 1960–2015. Then, the normalized time series in each observational estimate are offset by the difference of climatological means between observational estimate and **AR**. For example, the normalized time series of the regional averaged monthly burned area in GFED (y') are obtained from the following equation:

$$y' = \frac{\sigma_x^{short}}{\sigma_x^{all}} \frac{1}{\sigma_y^{short}} (y - \bar{\bar{y}}) + \bar{\bar{x}}'$$

where σ_x^{short} and σ_x^{all} are the standard deviations in **AR** for 1996–2015 and 1960–2015, σ_y^{short} the standard deviation in GFED for 1996–2015, y the monthly value in GWED, and $\bar{\bar{y}}$ and $\bar{\bar{x'}}$ the time means of y in GFED and of anomalies in **AR** (x') for 1996–2015, respectively. Consequently, the resulting time series in observational estimate have the same amplitude and climatological reference with **AR**. The NICC fire data are adjusted before and after 1983 separately because the NICC observations prior to 1983 include unknown sources.

Experiments

We conduct three sets of experiments using CESM: uninitialized (**UR**), assimilation (**AR**), and initialized runs (**IR**). In the 5-member ensemble simulations of **UR**, we prescribe natural and anthropogenic radiative forcings for the period of 1850–2100. Initial conditions for **UR** are obtained from 5 random years of a pre-industrial CESM control simulation. **UR** is used to adjust the model climatological biases relative to observation and to identify the externally forced

component (X_{ext}) and the internally generated climate variability (X_{int}). Estimates of the observed internal variability (X^o_{int}) added onto the model climatology (X^m_{clim}) and an estimate of the model externally forced signal (X^m_{ext}) are then assimilated back into the ocean model of CESM during an ensemble of coupled assimilation runs (i.e., the **AR)**. This method is similar to the anomaly assimilation conducted by previous studies[17,18] but takes into account the fact that the model externally forced component differs from the observation.

The separation into external and internal signals is achieved in the following way. Using the 3-dimensional temperature and salinity fields from the ECMWF ocean reanalysis product version 4 (ORA-S4)[19] as an observational estimate (X^o), we can decompose X^o into a mean climatology (X^o_{clim}, reference period: 1971-2000), an internal variability component (X^o_{int}) and an externally forced contribution (X^o_{ext}). Thus, we have $X^o = X^o_{clim} + X^o_{int} + X^o_{ext}$. To estimate ($X^o_{ext}$) we perform a Singular Value Decomposition (SVD) of the ORA-S4 temperature and salinity anomalies (calculated relative to the ORA-S4 climatology X^o_{clim} from 1971–2000) and anomalies of **UR** (calculated relative to the model climatology X^m_{clim} from 1971–2000) at each depth for the 1960–2005 period. The two fields of the leading SVD mode are then used as an estimate for X^o_{ext} and X^m_{ext} because the global warming trend associated with the external forcings is the dominant variation in the ensemble mean of **UR** as validated by previous studies[17,18].

The final 3-dimensional monthly ocean temperature and salinity fields obtained as $X^a = X^m_{clim} + X^o_{int} + X^m_{ext}$ (outside sea-ice regions) are then assimilated into the coupled climate model **AR** using the incremental analysis update scheme[20,21]. We apply strong and weak model-to-observation ratios (1/2 and 1/3, respectively) in analysis errors with a temporally and spatially invariant value and added analysis increments as forcing terms into the model's temperature and salinity tendency equations during an analysis interval of one day[18]. **AR** consists of 10 ensemble members with strong (5 members) and weak (5 members) model-to-observation ratios from January 1958 to June 2016. From **AR**, we first obtain a pair of atmospheric and oceanic initial conditions from the 10-member ensemble and then conduct a 10-year-long ensemble hindcast experiment, **IR**, starting from January 1st in every year from 1960 to 2016. The latest forecast of **IR** is initialized from January 1st, 2016 conditions. By assimilating anomalies relative to the model climatology, our **IR** has a negligible climate drift during the prediction period. Therefore, no post

processing for removing artificial drifts has to be applied to our initialized ensemble forecast simulations.

Supplementary Figure:

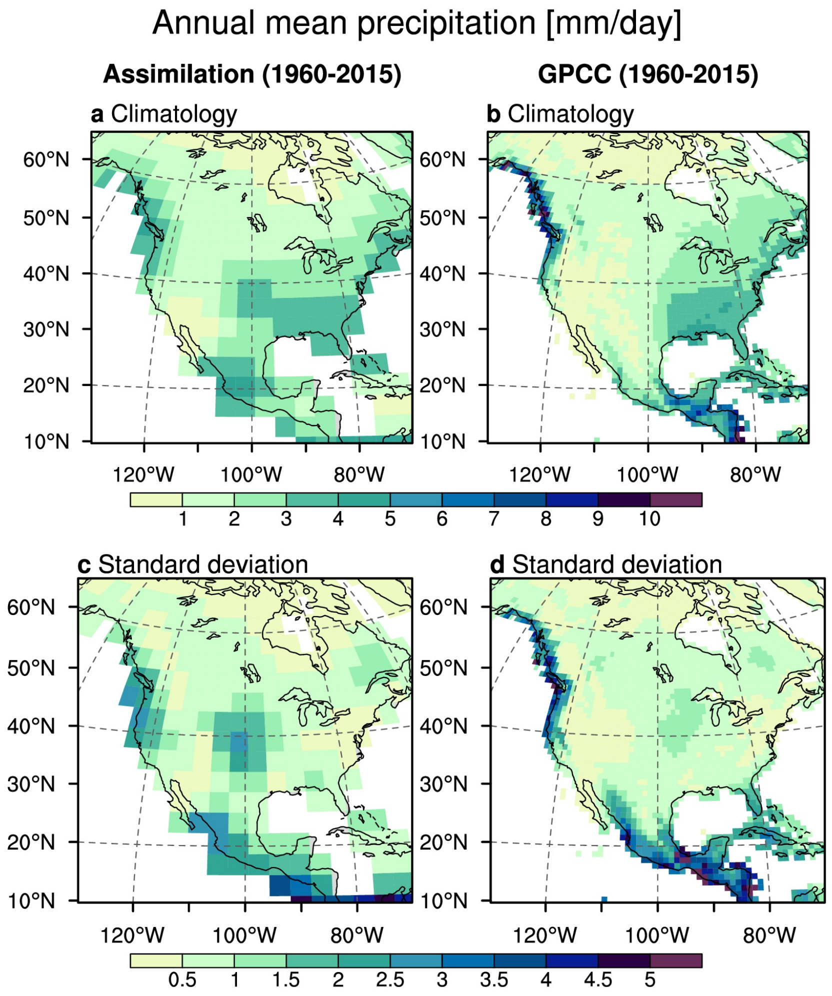

Fig. S1 Climatology (top) and standard deviation (bottom) for annual mean precipitation in assimilation **AR** (left) and observational estimate of GPCC [1] (right panel). Plots were generated using the NCAR Command Language (Version 6.3.0) [Software]. (2016). Boulder, Colorado: UCAR/NCAR/CISL/TDD. http://dx.doi.org/10.5065/D6WD3XH5.

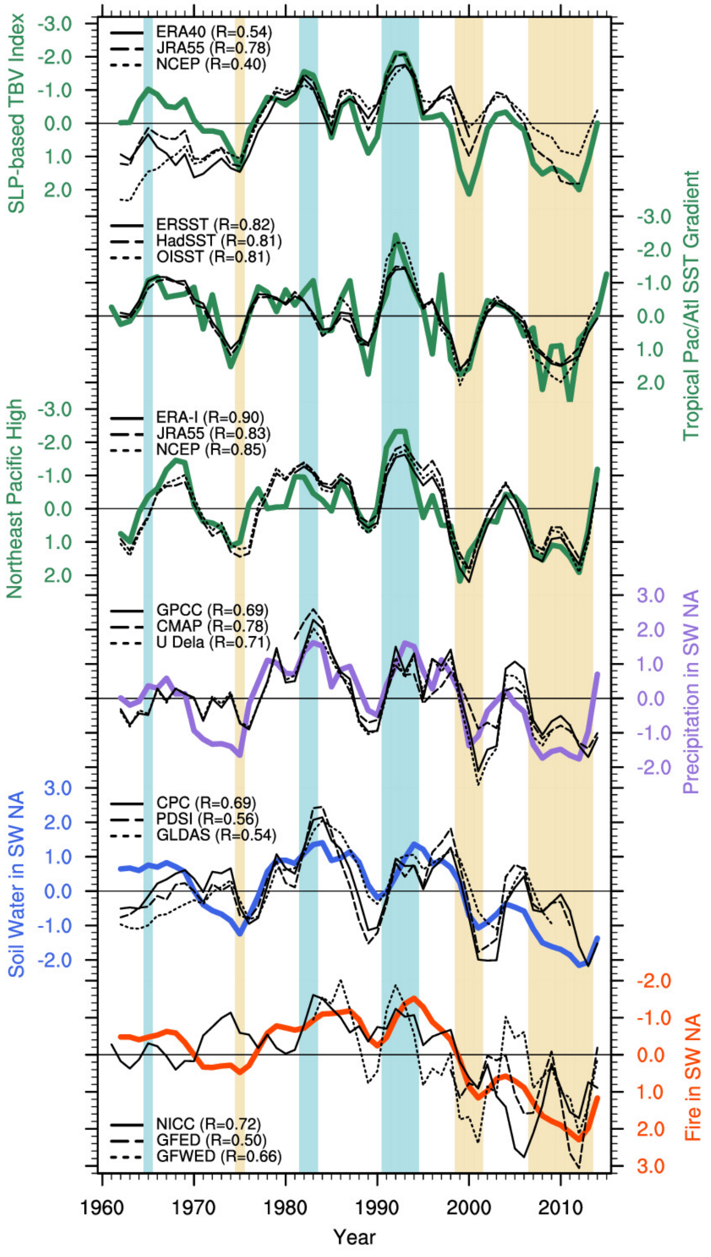

Fig. S2: Same as Fig. 1 but with 3-year running mean filter. Plots were generated using the NCAR Command Language (Version 6.3.0) [Software]. (2016). Boulder, Colorado: UCAR/NCAR/CISL/TDD. http://dx.doi.org/10.5065/D6WD3XH5.

First SVD mode (water year)

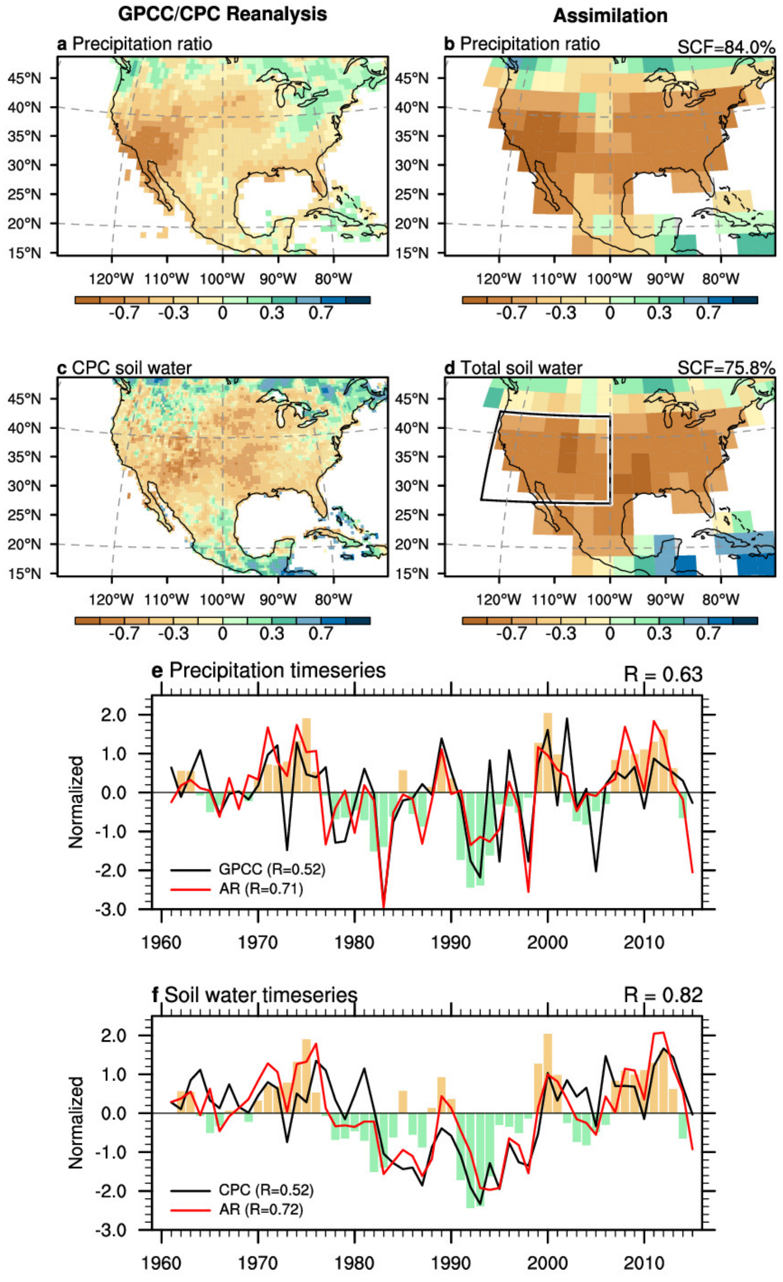

Fig. S3. Correlation patterns of annual mean precipitation ratio (top) and soil water anomalies (middle) associated with the first SVD modes between observational estimate (left; GPCC and CPC reanalysis) and assimilation (right). The box in (**d**) indicates the southwestern part of North America (28°N–44°N, 125°W–100°W). The squared covariance fraction explained by the first SVD mode is indicated in the upper-right corner of the right panels. The principal components of the first SVD modes of (**e**) precipitation ratio (their correlation coefficient R is 0.63) and (**f**) soil water anomalies (R=0.82) between observations (black) and assimilation run (red). Shadings in (**e, f**) correspond to the SLP-based trans-basin variability index (same with the top panel in Fig. 1). Correlation coefficients of the principal components in the first SVD mode with TBV index are shown in the bracket of (**e, f**). Plots were generated using the NCAR Command Language (Version 6.3.0) [Software]. (2016). Boulder, Colorado: UCAR/NCAR/CISL/TDD. http://dx.doi.org/10.5065/D6WD3XH5.

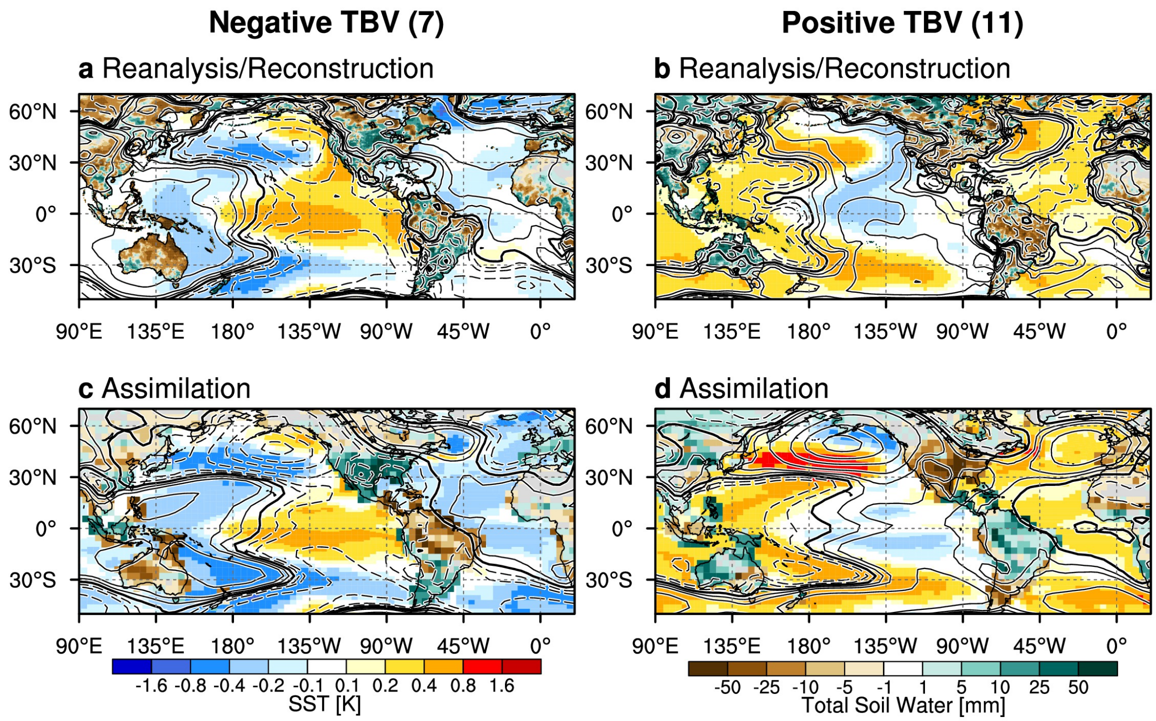

Fig. S4: Water year composite maps of SST (color in ocean), SLP (contour), and soil water anomalies (color in land) associated with 7 negative (left) and 11 positive years (right panels) of TBV phases in (**a**, **b**) observations, (**c**, **d**) CESM assimilation run. Observed SST, SLP, and soil water anomalies are obtained from ERSST[11], NCEP[9], and CPC datasets[2] as deviations from the 1960–2014 climatological means. These selected composite years correspond to exceeding +/- one standard deviation of the TBV index in Fig. 1. Negative contours are dashed and zero contours denote thick lines. Contour intervals for SLP anomalies are 0, ±0.1, ±0.2, ±0.5, ±1.0, ±1.5, ±2, and ±3 Pa. Plots were generated using the NCAR Command Language (Version 6.3.0) [Software]. (2016). Boulder, Colorado: UCAR/NCAR/CISL/TDD. http://dx.doi.org/10.5065/D6WD3XH5.

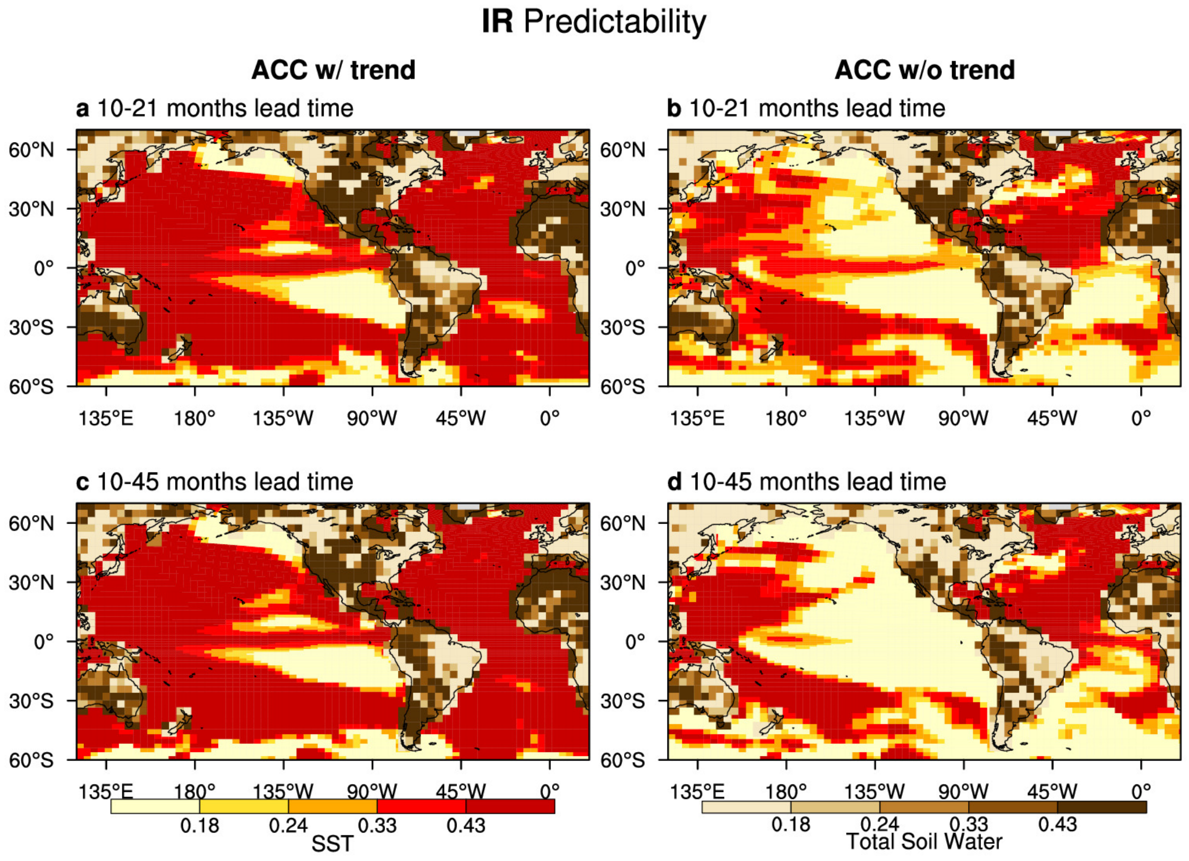

Fig. S5: Predictive skills of SST (ocean) and soil water anomalies (land) for averaged (top) 10–21 and (bottom) 10–45 months lead time in **IR** (left) with and (right) without 55-year long-term trends. In the right panels, the 55-year-long trends are linearly detrended in each **IR** and **AR**. Predictive skills in the **IR** are measured per grid-point by anomaly correlation coefficient against the **AR**. Correlation coefficients of 0.18, 0.24, 0.33 and 0.43 correspond to the statistical significance at 90, 95, 99 and 99.9% levels with 50 degrees of freedom on the basis of a one-sided Student's t-test. Plots were generated using the NCAR Command Language (Version 6.3.0) [Software]. (2016). Boulder, Colorado: UCAR/NCAR/CISL/TDD. http://dx.doi.org/10.5065/D6WD3XH5.

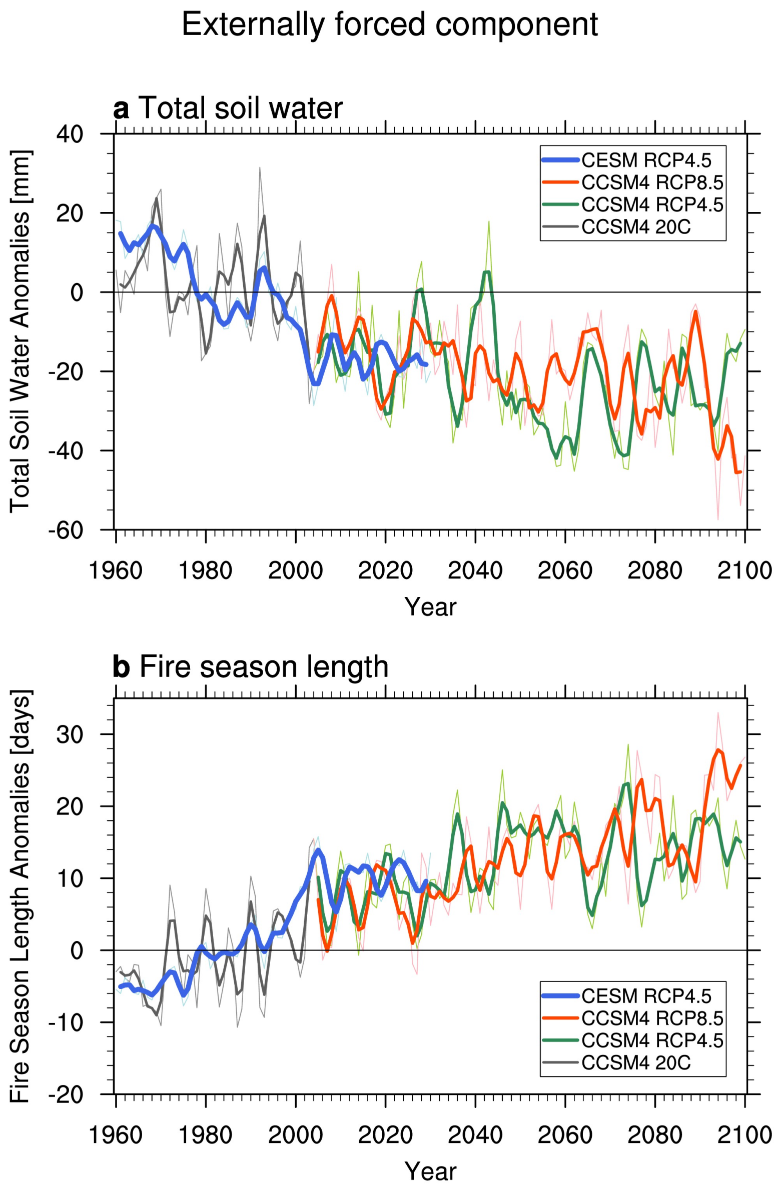

Fig. S6: Externally forced components of (**a**) total soil water and (**b**) fire season length anomalies over the Southwestern US for the 1960–2100 period. Externally forced components are estimated from the 10-member ensemble mean of CESM simulation prescribed with historical and RCP4.5 scenario forcings for 1960–2030 (CESM RCP4.5; blue) and the 5-member ensemble means of Community Climate System Model version 4 (CCSM4) simulations[22] prescribed with the historical forcings for 1960–2005 (CCSM4 20C; black) and the RCP4.5 (CCSM4 RCP4.5; green) and RCP8.5 scenario runs (CCSM4 RCP8.5; red line) for 2005–2100. Thin and thick lines are the annual mean and the 3-year running mean filtered time series, respectively. Plots were generated using the NCAR Command Language (Version 6.3.0) [Software]. (2016). Boulder, Colorado: UCAR/NCAR/CISL/TDD. http://dx.doi.org/10.5065/D6WD3XH5.

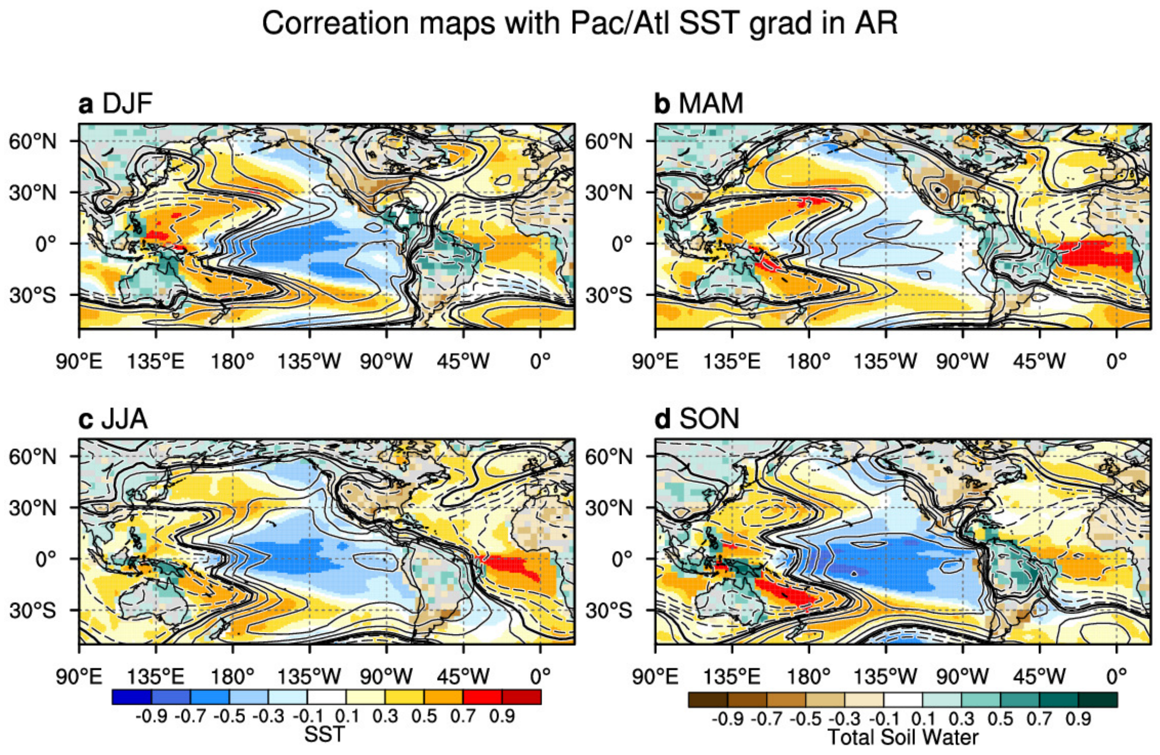

Fig. S7: Correlation maps of SST (color in ocean), SLP (contour), and soil water anomalies (color in land) with the tropical Pacific/Atlantic SST gradient during (**a**) DJF, (**b**) MAM, (**c**) JJA, and (**d**) SON seasons in **AR**. The SST gradient is defined as the zonal gradient of standardized SST anomalies between the tropical central Pacific (15°S–15°N, 180°–150°W) and the tropical Atlantic-Indian Ocean (15°S–15°N, 40°W–60°E). Contour intervals are 0, ±0.1, ±0.3, ±0.5, ±0.7, and ±0.9. Positive, negative and zero contours are thin, dashed and thick lines, respectively. Plots were generated using the NCAR Command Language (Version 6.3.0) [Software]. (2016). Boulder, Colorado: UCAR/NCAR/CISL/TDD. http://dx.doi.org/10.5065/D6WD3XH5.

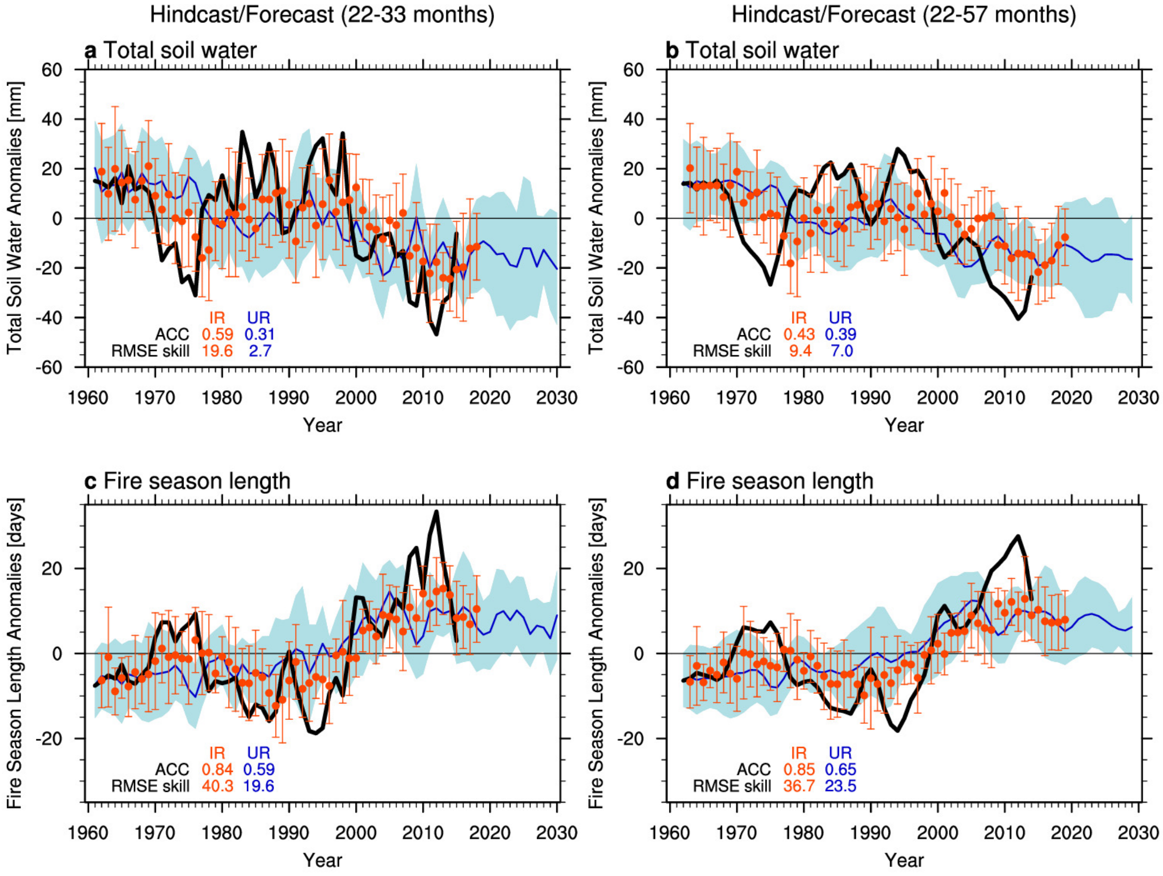

Fig. S8: Sane as Fig. 4 but for 22-33 and 22-57 months lead time for left and right panels, respectively. Plots were generated using the NCAR Command Language (Version 6.3.0) [Software]. (2016). Boulder, Colorado: UCAR/NCAR/CISL/TDD. http://dx.doi.org/10.5065/D6WD3XH5.

References in Supplementary Information

1 Schneider, U. *et al.* GPCC full data reanalysis version 6.0 at 0.5: monthly land-surface precipitation from rain-gauges built on GTS-based and historic data. (2011).

2 van den Dool, H., Huang, J. & Fan, Y. Performance and analysis of the constructed analogue method applied to US soil moisture over 1981-2001. *J Geophys Res-Atmos* **108** (2003).

3 Xie, P. & Arkin, P. A. Global precipitation: a 17-year monthly analysis based on gauge observations, satellite estimates, and numerical model outputs. *Bull. Amer. Meteorol. Soc.* **78**, 2539-2558 (1997).

4 Willmott, C. J., Matsuura, K. & Legates, D. Terrestrial air temperature and precipitation: Monthly and annual time series (1950-1999). *Center for climate research version* **1** (2001).
5 Dai, A., Trenberth, K. E. & Qian, T. T. A global dataset of Palmer Drought Severity Index for 1870-2002: Relationship with soil moisture and effects of surface warming. *Journal of Hydrometeorology* **5**, 1117-1130 (2004).
6 Rodell, M., Houser, P., Jambor, U. & Gottschalck, J. The global land data assimilation system. *Bulletin of the American Meteorological Society* **85**, 381 (2004).
7 Uppala, S. M. *et al.* The ERA-40 re-analysis. *Quarterly Journal of the Royal Meteorological Society* **131**, 2961-3012, doi:10.1256/Qj.04.176 (2005).
8 Ebita, A. *et al.* The Japanese 55-year Reanalysis "JRA-55" An Interim Report. *SOLA* **7**, 149-152, doi:10.2151/sola.2011-038 (2011).
9 Kalnay, E. *et al.* The NCEP/NCAR 40-year reanalysis project. *Bull. Amer. Meteorol. Soc.* **77**, 437-471 (1996).
10 Dee, D. P. *et al.* The ERA-Interim reanalysis: configuration and performance of the data assimilation system. *Quarterly Journal of the Royal Meteorological Society* **137**, 553-597, doi:10.1002/Qj.828 (2011).
11 Huang, B. *et al.* Extended reconstructed sea surface temperature version 4 (ERSST. v4). Part I: upgrades and intercomparisons. *Journal of Climate* **28**, 911-930 (2015).
12 Kennedy, J. J., Rayner, N. A., Smith, R. O., Parker, D. E. & Saunby, M. Reassessing biases and other uncertainties in sea surface temperature observations measured in situ since 1850: part 1. Measurement and sampling uncertainties. *J Geophys Res-Atmos* **116**, D14103, doi:10.1029/2010jd015218 (2011).
13 Reynolds, R. W., Rayner, N. A., Smith, T. M., Stokes, D. C. & Wang, W. An improved in situ and satellite SST analysis for Climate. *J. Climate* **15**, 1609-1625 (2002).
14 NICC. Wildland fire summary and statistics annual report 2016. (2016).
15 Giglio, L., Randerson, J. T. & Werf, G. R. Analysis of daily, monthly, and annual burned area using the fourth - generation global fire emissions database (GFED4). *Journal of Geophysical Research: Biogeosciences* **118**, 317-328 (2013).
16 Field, R. D. *et al.* Development of a Global Fire Weather Database. *Nat Hazard Earth Sys* **15**, 1407-1423, doi:10.5194/nhess-15-1407-2015 (2015).
17 Chikamoto, Y. *et al.* An overview of decadal climate predictability in a multi-model ensemble by climate model MIROC. *Climate Dyn.* **40**, 1201-1222 (2013).
18 Mochizuki, T. *et al.* Pacific Decadal Oscillation hindcasts relevant to near-term climate prediction. *Proc. Natl. Acad. Sci. USA* **107**, 1833 (2010).
19 Balmaseda, M. A., Mogensen, K. & Weaver, A. T. Evaluation of the ECMWF ocean reanalysis system ORAS4. *Quarterly Journal of the Royal Meteorological Society* **139**, 1132-1161, doi:Doi 10.1002/Qj.2063 (2013).
20 Bloom, S. C., Takacs, L., da Silva, A. M. & Ledvina, D. Data assimilation using Incremental Analysis Updates. *Mon. Wea. Rev.* **124**, 1256-1271 (1996).
21 Huang, B., Kinter, J. L. & Schopf, P. S. Ocean data assimilation using intermittent analyses and continuous model error correction. *Adv. Atmos. Sci.* **19**, 965-992 (2002).
22 Meehl, G. A. *et al.* Climate system response to external forcings and climate change projections in CCSM4. *Journal of Climate* **25**, 3661-3683 (2012).
